# Endoscopic Mucosal Resection Performed Underwater for Nonampullary Duodenal Epithelial Tumor: Evaluation of Feasibility and Safety

**DOI:** 10.1155/2018/7490961

**Published:** 2018-08-09

**Authors:** Goro Shibukawa, Atsushi Irisawa, Ai Sato, Yoko Abe, Akane Yamabe, Noriyuki Arakawa, Yusuke Takasaki, Takumi Maki, Yoshitsugu Yoshida, Ryo Igarashi, Shogo Yamamoto, Tsunehiko Ikeda, Hiroshi Hojo

**Affiliations:** ^1^Department of Gastroenterology, Aizu Medical Center, Fukushima Medical University, Aizuwakamatsu, Japan; ^2^Department of Pathology, Aizu Medical Center, Fukushima Medical University, Aizuwakamatsu, Japan

## Abstract

**Objectives:**

Recently, opportunities to encounter superficial nonampullary duodenal epithelial tumor (SNADET) have increased. EMR and ESD are performed to treat SNADET. However, the rate of perforation is higher than that of other gastrointestinal lesions, regardless of which method is used. Underwater EMR (UW-EMR) is immersion treatment of SNADET, which has low risk of perforation and can remove lesions safely and completely. In the present study, we retrospectively investigated patients in whom UW-EMR was performed to evaluate the feasibility and safety of UW-EMR for the treatment of SNADET.

**Methods:**

The primary endpoint was to evaluate the feasibility of UW-EMR for the treatment of SNADET, and secondary objective was to determine the operation's safety.

**Results:**

There were 14 participants, with a total of 16 lesions, who underwent UW-EMR between August 2015 and December 2017. Histological heteromorphism revealed that seven patients had low-grade adenoma, seven had high-grade adenoma, and two had adenocarcinoma. En bloc resection was performed in 14 lesions. In two patients, nodular lesions were observed in the scar and biopsy confirmed recurrences. There were no serious adverse events including bleeding or perforation.

**Conclusions:**

UW-EMR may be a safe and effective treatment method for SNADET, if its therapeutic indication is adequately considered.

## 1. Introduction

The incidence of all cancers of the small intestine, including superficial nonampullary duodenal epithelial tumor (SNADET), is remarkably lower than that of other gastrointestinal cancers, such as cancers of the stomach and large intestine [1]. The duodenum has more nonneoplastic lesions than other organs, and the incidence of malignancy among neoplastic lesions appears to be low. However, there has been a recent trend, starting in the 1970s, showing an increase in the incidence of malignancy among neoplastic lesions [2]. In addition, duodenal cancer is often detected at an advanced stage; the 5-year survival rate is less than 30%, and the prognosis is regarded as the worst among all small intestinal cancers [3].

In recent years, opportunities to encounter duodenal tumors have increased because of the popularization of endoscopy for upper gastrointestinal tract screening. However, no definite guidelines have been established regarding the indication of endoscopic therapy for duodenal tumor and selection of treatment strategy because of the low frequency of the procedure being performed.

Endoscopic mucosal resection (EMR) and endoscopic submucosal dissection (ESD) are performed to treat SNADET. However, because the duodenal muscularis propria is extremely thin, the rate of intraoperative or delayed perforation is higher than that of other gastrointestinal lesions, regardless of which method is used. Therefore, these techniques may not be suitable for use as standard treatments [4].

Binmoeller et al. [5, 6] developed the underwater EMR (UW-EMR), in which the EMR is performed while immersed in water. It has been reported that immersion treatment of a superficial tumor of the duodenum, which has a thin wall, and the large intestine, has an extremely low risk of perforation and lesions can be removed safely and completely. Because the muscular layer develops its weight by filling the lumen with water and the mucosal surface swells because of immersion, it is believed that the possibility of gripping the muscular layer at the time of snaring during EMR is extremely low. In the present study, we retrospectively investigated patients in whom UW-EMR was performed to evaluate the feasibility and safety of UW-EMR for treatment of SNADET.

## 2. Patients and Methods

### 2.1. Study Design

This was a retrospective study investigating the use of UW-EMR for treatment of SNADET. The primary endpoint was to evaluate the feasibility of UW-EMR for treatment of SNADET, and secondary objective was to determine the operation's safety. This study was reviewed and approved by the Institutional Review Board of Fukushima Medical University and conducted in accordance with the human and ethical principles of research set forth in the Declaration of Helsinki.

### 2.2. Patients

There were 14 participants, with a total of 16 lesions, who underwent UW-EMR in our department for the treatment of SNADETs between August 2015 and December 2017. The mean age of patients was 61.9 years, and the male to female ratio was 9 to 5. For treatment indication, irrespective of morphology (elevation/depression), the invasion depth was diagnosed endoscopically as an intramucosal tumor, and the size for inclusion was set to 20 mm or less. We set the exclusion criteria as follows: pregnancy, coagulopathy (international normalised ratio > 2.0, platelets < 70 × 100/L), previous endoscopic or surgical treatment of SNADET, and neoplastic lesions that do not meet the inclusion criteria.

### 2.3. Endoscopic Procedures

#### 2.3.1. Equipment

The endoscopes used were the GIF-HQ290, GIF-Q260, GIF-H290, and GIF-H290Z (Olympus Co., Tokyo, Japan), and the primary snare used was Captivator™ II (elliptical, 13 mm in diameter) (Boston Scientific Japan, Tokyo, Japan). However, depending on the size of the tumor to be treated, the Captivator™ II (elliptical, 27 mm in diameter) and Dualoop 33-16 (elliptical, 16 mm in diameter) (Medico's Hirata Inc., Osaka, Japan) or SD-7P-1 (semicircular, diameter of 23 mm) (Olympus Co., Tokyo, Japan) were used. VAIO 200S (ERBE Co. Ltd., Tuebingen, Germany) was used as the high-frequency generator, and the settings were as follows: Endocut-Q, effect 3, incision time two, incision interval three.

#### 2.3.2. Techniques

**Figure 1:**
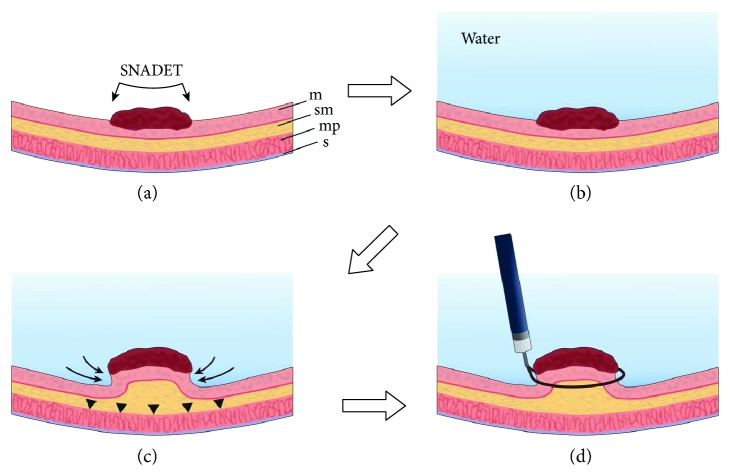
(a) The endoscope was inserted into the duodenum. m: mucosa, sm: submucosa, mp: muscularis propria, s: serosa. (b) After confirming the known tumor, slightly warm distilled water was injected into the duodenal lumen from the accessory channel, and the tumor was completely submerged. (c) The tumor was observed in underwater immersion and we observed until the lesion was slightly bulging from the mucosal surface (allow). During observation, the muscular layer was kept flat in a ring shape (allow head). (d) The snare was opened during underwater immersion and was subsequently pressed against the lesion site to confirm that the entire lesion entered the snare, and the lesion was strangulated. Resnaring was performed to confirm that the duodenal muscularis propria was not gripped, and the lesion was excised using an incision wave.

**Figure 2:**
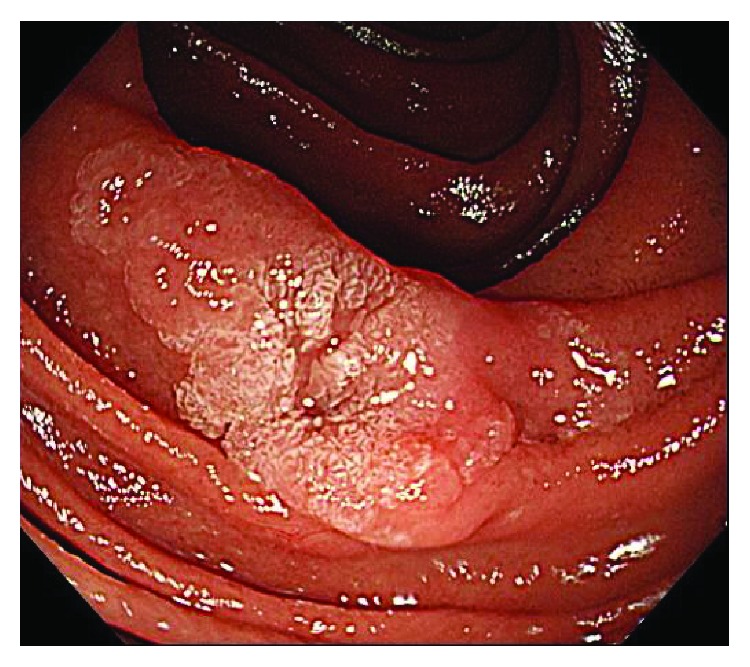
A slightly elevated lesion with a central irregular depression in the second part of duodenum was seen.

**Figure 3:**
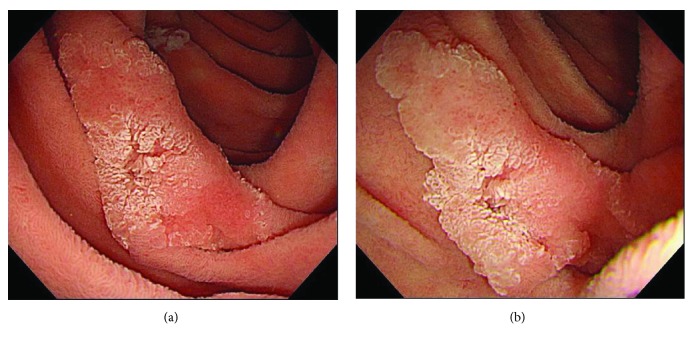
(a) Slightly warm distilled water was injected into the duodenal lumen from the accessory channel, and the tumor was completely submerged. (b) The lesion was slightly bulging from the mucosal surface a few minutes after warm water injection, and the boundary between the tumor and the normal mucosal surface became morphologically clear.

**Figure 4:**
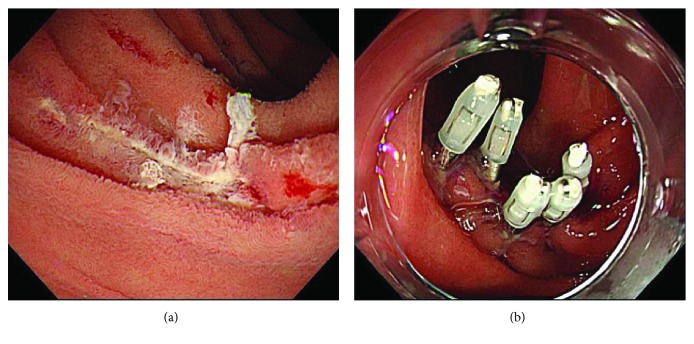
(a) The lesion was resected using a snare under water. (b) After resection, it was endoscopically confirmed that there was no residual tumor, and then the resected surface was stitched using a hemoclip.

UW-EMR was performed using the following procedures (schema was drawn as Figure 1):

1. The procedures were performed with the patient under sedation. Midazolam was used as the primary sedative (0.15 to 0.30 mg/kg was initially injected intravenously and, if necessary, half of the initial dose or the same amount was further administered), and when sedation was poor or the state of disinhibition due to midazolam use was noted, propofol (0.5 mg/kg/10 s) was also appropriately used in combination.
2. The endoscope was inserted into the duodenum with the patient in the left lateral decubitus position. After confirming the known tumor (Figure 2), slightly warm distilled water was injected into the duodenal lumen from the accessory channel, and the tumor was completely submerged (Figure 3(a)). If the stagnation of the distilled water in the duodenal lumen was poor when the patient was in the left lateral decubitus position, the position of the patient was changed to supine/abdominal position, so that favorable stagnation of distilled water could be obtained.
3. The tumor was observed in underwater immersion and we observed until the lesion was slightly bulging from the mucosal surface (Figure 3(b)).
4. The snare was opened during underwater immersion and was subsequently pressed against the lesion site to confirm that the entire lesion entered the snare, and the lesion was strangulated. Resnaring was performed to confirm that the duodenal muscularis propria was not gripped, and the lesion was excised using an incision wave (Figure 4(a)). Furthermore, the boundary between the tumor and the normal mucosal surface became morphologically clear because of the bulging of the lesion, and because the resected range was clearly observed together with the lens effect of the filled distilled water, preoperative marking was not performed.
5. After resection, the presence or absence of any remnant of the tumor was confirmed, and when the remnant was confirmed, additional resection was continued using UW-EMR.
6. After final resection, it was endoscopically confirmed that there were no remnants, and then the resected surface was stitched using a hemoclip (Figure 4(b)). If bleeding continued, even after clip plication, it was treated using cauterization by argon plasma coagulation (APC) and local injection of hypertonic saline epinephrine solution, as appropriate.
7. On the day after treatment, the presence or absence of an adverse event, such as hemorrhage, was confirmed by endoscopy. When no clear adverse events were present, eating was initiated on the second day following treatment. In all patients, proton pomp inhibitor was orally administered prior to the day on which the UW-EMR was performed.
8. Endoscopy was performed one to three months postoperatively for follow-up observation, and the healing process of the wound site was evaluated. We also assessed the existence of any remnants.

### 2.4. Assessment of Adverse Events

We predominantly assessed bleeding and perforation as adverse events related to this procedure. In terms of time performing this assessment, adverse events were classified as either intraprocedural or postprocedural. A definition was created with reference to ESGE's Colorectal polypectomy and EMR guideline [7] as follows: (1) Intraprocedural bleeding/perforation is bleeding/perforation occurring during the procedure that persists for more than 60 s or requires endoscopic intervention. (2) Postprocedural bleeding/perforation is bleeding/perforation occurring after the procedure, up to 30 days post-UW-EMR, that results in an unplanned medical presentation, such as emergency department visit, hospitalization, or reintervention (repeat endoscopy, angiography, or surgery). In addition, the degree of the adverse event was defined as follows: (1) A mild event involves slight bleeding where hemostasis could be achieved using an endoscopic procedure without a blood transfusion. (2) A severe event involves all bleeding, except the above-mentioned bleeding, and all perforation. Furthermore, we also evaluated the type and degree of relevant adverse events aside from bleeding and perforation.

## 3. Results

### 3.1. Feasibility (Table 1)

The sites of tumor occupation were as follows: duodenal bulb, one lesion (posterior wall, one lesion) and second part of duodenum, 15 lesions (anterior wall, two lesions; posterior wall, five lesions; left side wall, four lesions; right side wall, four lesions). Macroscopic findings indicated that 13 lesions were elevated and three were depressed.

The mean tumor diameter in the resected specimens was 10.5 mm (6–18 mm). Histological heteromorphism in the resected specimens revealed that seven patients had low-grade adenoma, seven had high-grade adenoma, and two had adenocarcinoma. The invasion depth of adenocarcinoma was mucosal carcinoma in all patients. Evaluation of the lateral stump of the tumor in the resected specimens revealed various results. One stump tested positive for high-grade adenoma. In three lesions, the stump was diagnosed as negative for low-grade adenoma. However, evaluation of the other 12 lesions was difficult. En bloc resection was performed in 14 lesions (87.5%), but the remaining two lesions (12.5%) showed a remnant after initial the UW-EMR, and we performed additional resection on the same day. Fractional excision in two parts was performed for one lesion located on the left side wall of the second part of duodenum, and fractional excision in four parts was performed for one lesion located on the posterior wall of the second part of duodenum.

Endoscopic examination for follow-up observation was performed one to three months postoperatively (one patient received their endoscopic examination for follow-up observation one year postoperative because their postoperative diagnosis was low-grade adenoma). In two patients, small nodular lesions were observed in the UW-EMR scar. In case number 1 (second part of duodenum, posterior wall), a nodule was found in the scarred part when the endoscopy was performed for follow-up observation one month later, and adenocarcinoma was confirmed by biopsy in the same part (pathological diagnosis of resected specimen was adenocarcinoma). Fractional excision in four parts was performed in the present patient. However, in case number 12 (second part of duodenum, left side wall), en bloc resection was performed, but the results of the follow-up endoscopy performed six months after treatment revealed the presence of a white nodule in the scarred part, and a biopsy confirmed high-grade adenoma in the same part (pathological diagnosis of resected specimen revealed high-grade adenoma). In all cases, because the lesion was present in the scar after resection, additional endoscopic treatment was difficult and surgical resection was performed. In the 14 additional lesions, remnant recurrence during the mean observation period of 10.8 months (1–28 months) was not noted.

## 4. Safety (Table 1)

With regard to adverse events, a minor intraprocedural event (minimal oozing of blood) was observed in three patients (case number 8, 12, and 13) and a minor postprocedural event (minimal oozing of the blood) in one (case number 9), but there were no serious adverse events, such as bleeding or perforation, that required blood transfusion. In the two patients who experienced an intraprocedural event, hemostasis was achieved by performing plication of the wound with a clip, and in the other patient, because minor oozing of blood was observed after wound plication, hypertonic saline-epinephrine solution was injected locally into the hemorrhage site to stop the bleeding. In one patient with postprocedural bleeding, minor oozing of blood from the wound was observed during the endoscopic observation performed the day following operative treatment, and hemostasis was achieved using APC cauterization.

## 5. Case Presentation

**Figure 5:**
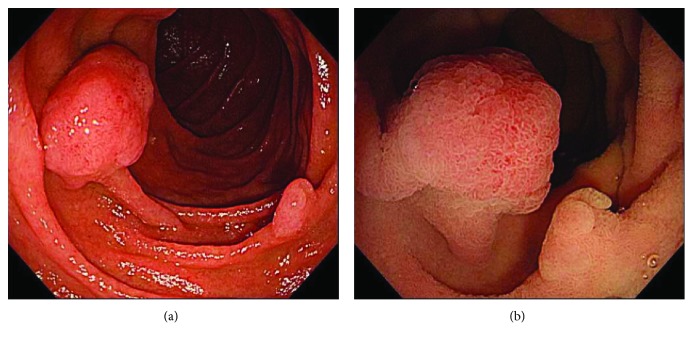
(a) There was an elevated lesion on both the left side and the right wall of the second part of duodenum. (b) The boundary between the tumor and the normal mucosal surface became morphologically clear in underwater immersion.

**Figure 6:**
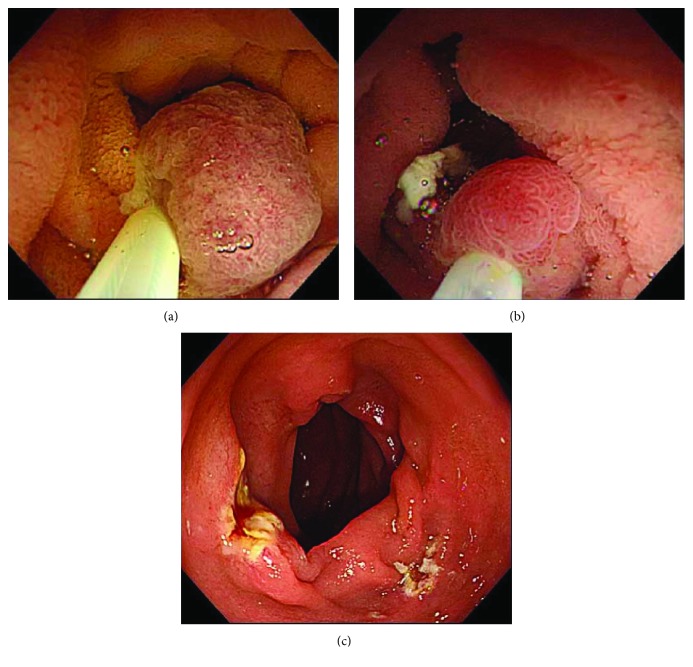
(a, b) Two lesions were collectively excised using UW-EMR. (c) Minimal oozing of blood was seen after UW-EMR.

**Figure 7:**
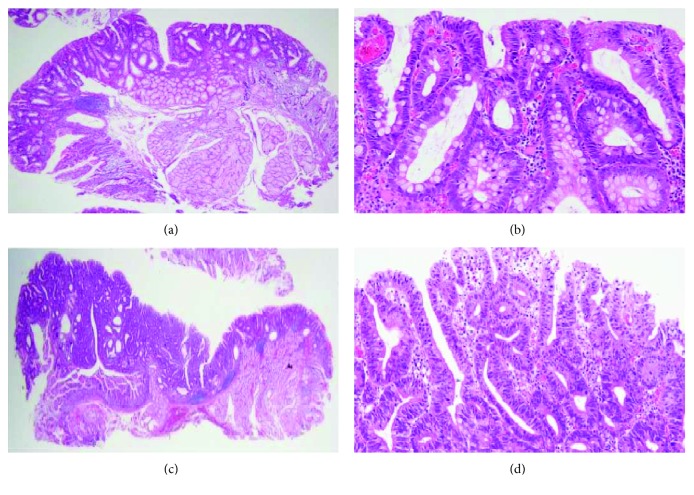
(a, b) Histopathological examination for larger lesion revealed that lesions in which adenocarcinoma was suspected during preoperative biopsy were revealed to be adenocarcinoma (HE staining, ×40 in (a), ×200 in (b)). (c, d) Histopathological examination for smaller lesion diagnosed as adenoma (HE staining, ×40 in (c), ×200 in (d)).

**Figure 8:**
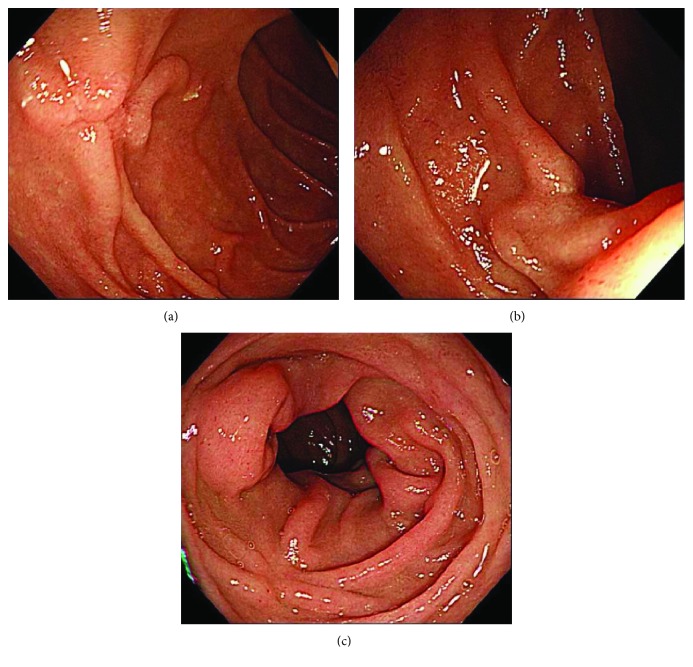
(a, b) Follow-up endoscopic examination, which was performed one month after the UW-EMR, showed wound scarring in both lesions without recurrence. (c) Endoscopic examination at 21 months after treatment showed no recurrence.

The patient was a 60-year-old woman (case number 3 on Table 1). There was one elevated lesion on both the left side wall and the right wall of the second part of duodenum (Figure 5), and because biopsy findings were suspected of adenocarcinoma, the patient visited our department for treatment. Along with small lesions for which biopsy was not performed, two lesions were collectively excised using UW-EMR (Figure 6). Four clips were used on the resected surface and one for plication. The postoperative course was favorable, and complications such as bleeding and perforation were not observed, even after resuming eating. She was discharged on postoperative day eight. Lesions in which adenocarcinoma was suspected during preoperative biopsy were revealed to be adenocarcinoma by the final pathological diagnosis of the resected specimens (Figures 7(a) and 7(b)), and the final pathological diagnosis of the other lesions was adenoma (Figures 7(c) and 7(d)). Follow-up endoscopic examination, which was performed one month after the UW-EMR, showed wound scarring in both lesions, and endoscopic findings suggesting recurrence were not observed (Figures 8(a) and 8(b)). Endoscopic examination subsequently performed at 6, 12, and 21 months after treatment revealed no recurrence (Figure 8(c)).

## 6. Discussion

ESD and EMR have been performed as endoscopic treatment of SNADET. In facilities with an experienced endoscopist, the risk of complications related to EMR is low and the procedure can be safely performed [8]. However, in the case of a lesion with a diameter of 20 mm or larger, there is a high possibility that fractional excision is performed in EMR, making it difficult to perform a pathological assessment after resection, and recurrence of the remnant after resection is a problem. In ESD, en bloc resection is possible for such large lesions, but because the duodenal muscularis propria is extremely thin, the intraoperative and delayed perforation rates are high [4]. Binmoeller et al. [5, 6] reported that by observing the tumor in underwater immersion in the UW-EMR, the deep muscular layer is kept flat in a ring shape, and the mucosa and submucosal layer of the tumor rise to the side of the intestinal lumen, filled with injected water so as to float from the muscular layer. Therefore, it is possible to grip the lesion with a snare without gripping the muscular layer. In addition, observation by insufflation revealed that the lesion part was extended and stretched along with the intestinal wall, and it is difficult to simply grip using the snare only. But, in the UW-EMR, the gastrointestinal wall of the lesion is in a relaxed state, so that gripping by snare becomes easier and a wider range of the lesion is available to be gripped, as compared to the use of a conventional EMR. In the present study, of the 14 patients (16 lesions) who underwent treatment, one lesion was present in the duodenal bulb and 15 lesions were present in the second part of the duodenum. Furthermore, these lesions were distributed in various parts of the second part of the duodenum. Of the target lesions in the present study, fractional excision had to be performed in two, but it was demonstrated that this method can be performed safely without causing serious complications up to the second part of the duodenum. Although clear guidelines for lesions indicated for treatment are needed in the future, this method may become a first-line therapy for SNADET.

Binmoeller et al. [5], who initially reported this method, regarded adenoma as the indication for UW-EMR. Therefore, even for laterally spreading duodenal adenoma with a large tumor diameter, treatment with fractional excision by this method was performed. By contrast, because we also regarded adenocarcinoma as an indication, considering the risk of remnant and pathological evaluation, lesions large enough to undergo en bloc resection (in principle, lesions less than 20 mm that can be gripped by snare) were indicated for treatment. Cases requiring planned fractional excision were excluded from the indication. As a result, when the tumor diameter was 20 mm or less, en bloc resection was possible in 87.5% of cases. However, en bloc resection could not be performed in two lesions (12.5%), and additional resection was performed. The sizes of these lesions were 15 mm and 13 mm, and even when compared to other lesions, their size allowed en bloc resection to be sufficiently performed. The two lesions in which fractional excision was performed were not particularly unique tumor sites in comparison with other lesions, but due to the curve of the duodenum and positional relation with the duodenal folds, it is possible that the identification of the area on the anal side was difficult, or alignment of the snare could not be sufficiently controlled. From this viewpoint, it appears necessary to attempt using a snare ring for under water immersion before treatment with this method. There are also various kinds of snares (e.g., round, oval, or semicircular shape; hard or soft material), and the selection of the device according to the size and lesion site is also important. It is the authors' opinion that, in any case, it is extremely important to establish a proper strategy before surgery.

In the patients in the present study in whom the procedure was performed, evaluation of the lateral stump was possible in only four lesions, and it was difficult to evaluate the other 12 lesions. The reason for the difficulty in assessment in UW-EMRs appears to be related to the influence of thermal denaturation on the resected side surface by gripping the mucosal surface in an unexpanded state with the snare and performing the excision. Although it was endoscopically confirmed that there was no remnant at the time of the UW-EMR, careful follow-up observation, such as shortening of the observation period, may be necessary when the lateral stump in the resected specimen is difficult to evaluate. In fact, in the present study, we experienced recurrence in the remnant in two lesions (two patients). One lesion was adenocarcinoma for which fractional excision in four parts was performed. Because fractional excision seems to have a high risk of remnants, it is necessary to establish a solid strategy for collective resection for treatment, as previously mentioned. In cases where it is difficult to perform an en bloc resection, another treatment method should be considered without overly focusing on this method. However, in another lesion in which recurrence was noted, en bloc resection could be performed, but a nodule was confirmed in the scarred part of UW-EMR by endoscopy one month after treatment and a remnant was confirmed. Because this lesion was located in a site lateral to the superior duodenal angle where the duodenum had a large curve, observation of the anal side margin of the lesion could not be sufficiently performed, and snaring was performed in a state in which all lesions were not observed. When the entire image of the lesion is difficult to observe in one field of view, caution is needed with regard to the risk of the remaining remnants, and it is necessary to consider a treatment strategy without overly focusing on this method.

In the present study, 15 out of 16 lesions were preoperatively diagnosed by endoscopic biopsy, and the concordance rate of preoperative diagnosis and postoperative pathological diagnosis was 60% (9 out of 15 lesions). The problem of diagnostic accuracy of preoperative biopsy for SNADET has been previously indicated, and Goda et al. [9] and Kakushima et al. [10] reported a low proper diagnosis rate (68% and 74%, resp.). The appropriate diagnosis rate of preoperative diagnosis by endoscopy was higher (75% and 78%, resp.), which was due to recent improvements in endoscopic diagnostics and the development of diagnostic techniques using imaging enhancement, such as NBI and magnifying endoscopy. However, there is no established consensus regarding the criteria for endoscopic diagnosis of SNADET, and the fact that there are variations in diagnosis by surgeon and institution remains a problem. Of the types of SNADET, low-grade adenoma is also thought to be a lesion for which follow-up is possible [11], but at present, the proper diagnosis rate in endoscopic diagnosis and pathological diagnosis is low, and the possibility of malignancy cannot be denied, so it may be difficult to make the determination that follow-up is possible. Therefore, as the precision of preoperative diagnosis is low, endoscopic treatment may also be considered as a diagnostic treatment. In such a case, ESD, which is at a high risk of severe adverse event, cannot be used as a standard diagnostic treatment. However, in the present study, the safety of UW-EMR was strongly demonstrated, and from this viewpoint, there is a possibility that it may become a standard treatment of SNADET. Endoscopic treatment for SNADET, including nonmalignant tumors especially such as adenoma, needs to be minimally invasive, and even with the ease of the procedure and the low risk of adverse events, UW-EMR may be a standard treatment for adenoma.

In conclusion, UW-EMR may be a feasible treatment method for SNADET, if its therapeutic indication is adequately considered. It is the authors' opinion that, especially in the case of adenoma with a tumor diameter less than 20 mm, it may be beneficial to actively consider the procedure. However, because the present study was both a retrospective and small case series study, to establish the utility, effectiveness, and safety of UW-EMR for treating SNADET in the future, it is necessary to conduct a prospective comparison study with ESD.

## Figures and Tables

**Table 1:** Patient characteristics and therapeutic summary.

Case	Location	Macroscopic type	Size of snare	Biopsy diagnosis	Tumor size	Final diagnosis	Histological margin	En bloc or multiple resection	Recurrence (follow-up period)	Adverse event
1	2nd	Protuberance	20 mm	Group 4	13 mm	Aca	D/E	Multiple (4)	Recurrence (1 M)	(−)
2	2nd	Protuberance	27 mm	Adenoma	12 mm	LGA	D/E	En bloc	No (28 M)	(−)
3	2nd/2nd	Protuberance/protuberance	13 mm	Aca/undone	12 mm/6 mm	Aca/LGA	D/E/D/E	En bloc/En bloc	No (27 M)/No (27 M)	(−)/(−)
4	2nd	Protuberance	13 mm	Adenoma	11 mm	HGA	D/E	En bloc	OFD (10 M)	(−)
5	2nd	Protuberance	13 mm	Adenoma	9 mm	LGA	pHM0	En bloc	No (13 M)	(−)
6	2nd	Protuberance	13 mm	Group 4	8 mm	HGA	D/E	En bloc	No (12 M)	(−)
7	2nd	Protuberance	13 mm	Adenoma	10 mm	HGA	D/E	En bloc	No (12 M)	(−)
8	Bulb	Protuberance	13 mm	Hyperplastic	15 mm	LGA	D/E	En bloc	No (10 M)	Bleeding during UW-EMR
9	2nd	Protuberance	16 mm	Adenoma	15 mm	LGA	D/E	Multiple (2)	No (10 M)	Bleeding post-UW-EMR
10	2nd	Protuberance	13 mm	Adenoma	12 mm	HGA	D/E	En bloc	No (10 M)	(−)
11	2nd/2nd	Depress/depress	13 mm	Adenoma/HGA	7 mm/6 mm	LGA/HGA	pHM0/D/E	En bloc/En bloc	No (5 M)/No (5 M)	(−)/(−)
12	2nd	Protuberance	18 mm	Adenoma	8 mm	HGA	D/E	En bloc	Recurrence (1 M)	Bleeding during UW-EMR
13	2nd	Protuberance	13 mm	Adenoma	6 mm	HGA	pHM1	En bloc	No (1 M)	Bleeding during UW-EMR
14	2nd	Depress	13 mm	Adenoma	18 mm	LGA	pHM0	En bloc	No (1 M)	(−)

Aca: adenocarcinoma, LGA: low-grade adenoma, HGA: high-grade adenoma, D/E: difficulty of evaluation, OFD: other factors death.

## Data Availability

The data used to support the findings of this study are available from the corresponding author upon request.
